# Proteomics and Metaproteomics Add Functional, Taxonomic and Biomass Dimensions to Modeling the Ecosystem at the Mucosal-luminal Interface

**DOI:** 10.1074/mcp.R120.002051

**Published:** 2020-11-25

**Authors:** Leyuan Li, Daniel Figeys

**Affiliations:** Department of Biochemistry, Microbiology and Immunology, Ottawa Institute of Systems Biology, Faculty of Medicine, University of Ottawa, Ottawa, Canada

**Keywords:** Microbiome, mathematical modeling, bacteria, gastrointestinal disease, exosomes, viruses, ecology, host-microbiome interaction, metaproteomics, proteomics

## Abstract

Recent efforts in gut microbiome studies have highlighted the importance of explicitly describing the ecological processes beyond correlative analysis. However, we are still at the early stage of understanding the organizational principles of the gut ecosystem, partially because of the limited information provided by currently used analytical tools in ecological modeling practices. Proteomics and metaproteomics can provide a number of insights for ecological studies, including biomass, matter and energy flow, and functional diversity. In this Mini Review, we discuss proteomics and metaproteomics-based experimental strategies that can contribute to studying the ecology, in particular at the mucosal-luminal interface (MLI) where the direct host-microbiome interaction happens. These strategies include isolation protocols for different MLI components, enrichment methods to obtain designated array of proteins, probing for specific pathways, and isotopic labeling for tracking nutrient flow. Integration of these technologies can generate spatiotemporal and site-specific biological information that supports mathematical modeling of the ecosystem at the MLI.

## Teaser

Proteomics and metaproteomics are important tools for studying the spatiotemporal heterogeneous ecosystem in our gut. We review strategies and their applications to gut ecology studies, such as building a dynamical model of the MLI.

## Graphical Abstract

**Figure:**
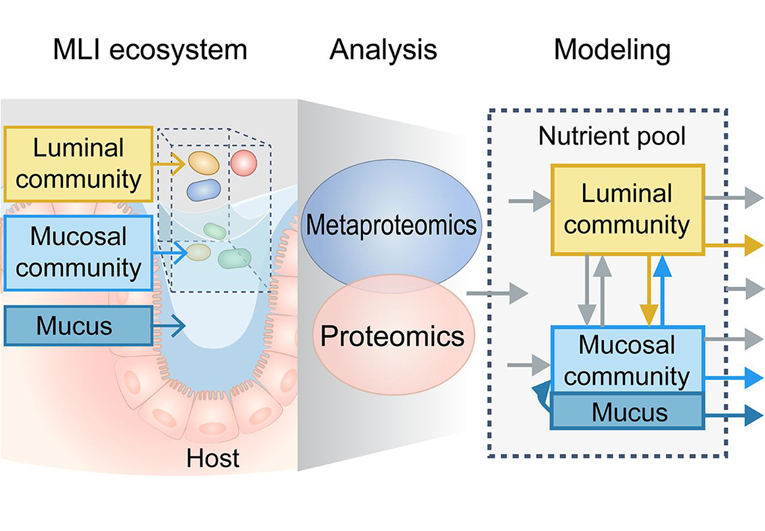

• The gut mucosal-luminal interface is a spatiotemporal heterogeneous ecosystem.
• Proteomics and metaproteomics are tools to study the host and microbiome functionality.
• Insights into functional diversity, biomass, and matter flow can be obtained.
• Such data can be complementary inputs for building ecology models of the microbiome.

The human mucosal-luminal interface (MLI) is a complicated ecosystem where interactions between the mucosal and luminal communities, as well as between host and microbiome take place (1, 2). Longitudinal surveys showed that healthy individual gut microbiomes are dynamically stable over time (3, 4). The ecological principles behind the maintenance of microbiome diversity, stability and host-microbiome homeostasis remains largely unexplored. A better understanding of these ecological principles might lead to novel approaches to treat diseases.

The MLI possesses strong spatial and temporal heterogeneity, forming different niches along both longitudinal and transverse axes (5) as well as displaying circadian rhythmicity (6). Niche partitioning results in diversity, structural and functional variability of microbial communities, and it is also a factor contributing to dynamically stable coexistence between these communities. Therefore, understanding the ecological mechanisms behind host-microbiome homeostasis requires characterization of the gut microbiome and the host factors according to the spatiotemporal attributes, which largely relies on a proper selection of analytical tools.

Studying the functional ecology at the MLI can benefit from the use of different analytical approaches, such as high throughput-omics techniques. Compared with metagenomics and metatranscriptomics, proteomics and metaproteomics can provide additional valuable insights, including biomass, matter and energy flow, and functional expressions. In this Mini Review, we narrow down the topic from the broad sense of ‘ecology’ to the more specific discipline of theoretical ecology that uses models and simulations to study the community diversity, functionality, interaction, and dynamics of an ecosystem. We discuss questions that are involved in studying the MLI ecology in this scope, and review proteomics and metaproteomics methodologies that can generate adequate arrays of data for such studies.

## PROTEOMICS AND METAPROTEOMICS TECHNIQUES AT a GLANCE

Proteomics identifies and quantifies proteins in a single-species sample, *e.g.* cells, tissues, secreted host proteome in stool, etc. Metaproteomics extends proteomics to study a multi-species microbial community, *e.g.* gut microbiome. Currently, most proteomics and metaproteomics approaches are based on liquid chromatography coupled to tandem MS (LC–MS/MS). In a typical proteomic analysis, a complex mixture of peptides, as a result of proteolytic enzyme digestion of a protein extract, is separated by LC and introduced to the MS, where peptides ions are separated based on mass/charge (*m*/*z*). With data-dependent acquisition (DDA), top N precursors are selected for fragmentation, and the resulting MS/MS spectra are then assigned to peptide sequences by database searching. More technical details have been reviewed by other researchers (7, 8). More recently, data-independent acquisition (DIA) has been developed and demonstrated as a promising approach in proteomics as well as metaproteomics, which improved reproducibility of peptide quantification between technical replicates, as well as proportion of shared peptides between different samples (9). Compared with classical proteomics, metaproteomics is more challenging in many aspects, including the higher sample complexity, larger size of database, and more complicated data processing/analysis. Nevertheless, the continuous evolutions of proteomics and metaproteomics have made a powerful impact on how we could use them to understand the host-microbiome ecology.

## STRUCTURE AND ECOLOGY OF THE MLI

**Fig. 1:**
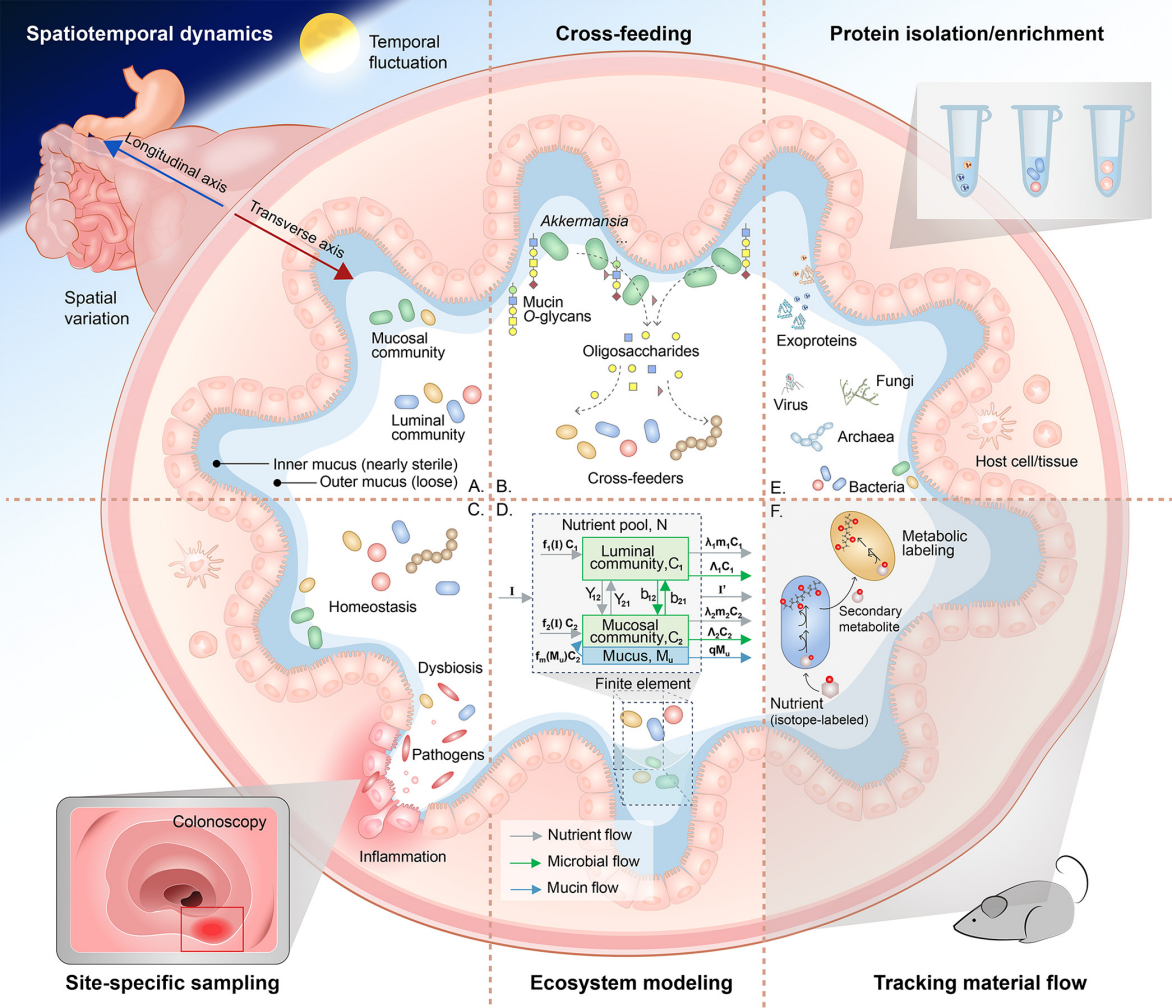
Structure and ecology of the MLI. *A*, Spatiotemporal dynamics of the MLI ecosystem. *B*, Cross-feeding mechanism between intestinal species: an example of *Akkermansia*. *C*, Comparison between host-microbiome homeostasis and dysbiosis. Site-specific sampling can be performed using colonoscopy. *D*, An example of studying the MLI using mathematical modeling approach (see supplemental Information for details). *E*, Different biological components of the MLI can be extracted using a series of isolation/enrichment techniques for proteomics/metarpteomics analysis. *F*, Tracking material flow can be achieved by combining metaproteomics with metabolic labeling approaches.

The gastrointestinal tract is spatially heterogeneous along both the longitudinal and cross-sectional axes. Levels of oxygen, pH, nutrient, and host immune activity vary along the longitudinal axis (5, 10). Along the transverse axis, the epithelial surface of the colon is coated with two different layers of mucus: the inner layer is firmly attached and is relatively sterile, providing a barrier for microbial invasion to the host; the outer layer is loose and harbors a diverse population of mucosal commensals (11). The gut mucus layer is composed of host-secreted mucin *O*-glycans (12), whereas nutrients in the gut lumen are present in the form of passing food bolus. Because of the niche differences, the mucosal and luminal portions harbor microbial communities that are distinct in composition, diversity, species abundance distributions (13, 14, 15). The mucus layer is featured with higher abundances of Firmicutes and the luminal contents are enriched in Bacteroidetes (15). Mucosal and luminal communities show different responses to nutrient or other compounds that pass through the intestine (14, 16) (Fig. 1*A*). In addition to spatial organizations, temporal change of the MLI is also a contributor to gut homeostasis. Host anti-microbial peptides, glucocorticoid hormones and mucus secretion are influenced by the circadian rhythm. And mucosal-adherent bacteria also show diurnal oscillations in composition and function (6).

Only a subset of gut commensals can use mucin glycans in the mucus. *Akkermansia* is a major mucin degrader that can breakdown mucin glycosidic chains through extracellular β-galactosidases, and liberate oligosaccharides that are accessible for other members of the microbiome (17, 18, 19). In addition, *Bifidobacteria* can degrade dietary and host-produced glycans, and subsequent cross-feeding mechanisms enhanced formation of butyrate by other gut microbes (20, 21, 22). Under the condition of limited types of available nutrients and low amount of free short-chain carbohydrates in the gut, bacteria cross-feeding mechanisms (Fig. 1*B*) play important roles in maintaining microbiome diversity and dynamic stability.

The host also plays an important role in maintaining the gut homeostasis. On one hand, the host secrets nutrients, such as mucin glycans, to harbor a reservoir of gut symbionts; on the other hand, it possesses mucosal surfaces that serve as a first line of defense against bacterial attack. The host immune system plays a crucial role in maintaining homeostasis at mucosal surfaces. The host resists invasion of pathogens partially through the expression of antimicrobial proteins and peptides regulated by cytokines of the innate and adaptive immune systems (23). In addition to the host's role in pathogen clearance, adherence of commensal bacteria can be selectively promoted by IgA (IgA) antibody (5). Such activities facilitate stable colonization of particular mucosal niches and exclude exogenous competitors. Compromised mucosal barrier and inappropriate immune activation by commensals mislocalized to the mucosa is associated with diseases such as inflammatory bowel diseases (IBD) (24). Dysbiosis in IBD is observed with an increase in Proteobacteria and a decrease in Firmicutes (25) (Fig. 1*C*).

Ecosystem modeling of the microbe-microbe and host-microbiome interactions at the MLI will provide a theoretical framework for gut microbiome-related health and disease. Classical ecological theories and hypothesis (26) are worth referring for understanding the biodiversity, stable co-existence of species and their relationship with the spatiotemporal properties of the MLI. Compared with conventional environmental ecology studies, fewer ecological hypothesis have been examined in our gut ecosystem. The MLI possesses strong spatial heterogeneity, and spatial structure (nutrient niches) is an important factor for stable coexistence of species (27), because it allows the species to best adapt to particular environmental conditions and make best use of the available resources (28, 29). Verster *et al.* examined whether the competitive lottery model (a niche colonization theory) established for macro communities could apply to the gut microbiome. The study discovered lottery-like assembly pattern of bacterial species that are more functionally specialized than other members of the microbiome (30). Organisms also tend to generate clusters of conspecifics, which also increases the potential for coexistence and maintains the biodiversity (26). Another interesting question is how the gut diurnal rhythm contributes to MLI homeostasis. Niche theories may still be insufficient to explain the stable coexistence of the highly diverse gut species, because there are far less types of nutrients than the number of species in the gut. Shaani *et al.* showed that environmental change, such as food intake, could induce microbial niche modification and subsequently drive diurnal community assembly (31). A “nonequilibrium coexistence of competitors” theory states that temporal changes of environment may provide alternative competitive advantages to different species (32), which potentially could explain the stability and diversity of the microbiome.

## IMPORTANCE OF PROTEOMICS AND METAPROTEOMICS IN MLI STUDIES

With ecological questions largely unexplored, it is crucial to select proper analytical tools to generate adequate data set for studying the MLI ecosystem. Current studies on the mucosal-luminal interface use numerous tools, including flow cytometry (33), 16S rRNA gene PCR amplification (16, 34, 35), qPCR (34, 35), transcriptomics (34, 35), contig‐based viral genotype profiling (36), morphological analysis (10, 33) and proteomics/metaproteomics (37, 38, 39, 40). Finally, a comprehensive ecological systems biology approach is required to integrate theory and experiments to unveiling the complex MLI ecology. Among these tools, proteomics and metaproteomics are important experimental approaches for MLI ecology studies, for the following reasons:

##### Functional Diversity is an Important Dimension of Biodiversity

Recent research focus of community ecology has extended from explaining species diversity to elucidating the functional dimension of biodiversity (26). Because of the complexity of functional capacity in different members of the microbiome, several recent studies have clustered microbial species into groups based on their ecological niches and component functional attributes, *i.e.* functional guilds (41). Genome sequences represent functional potentials that are not representative of protein levels (42), and RNA expression have limited correlation to the actual abundance of proteins (43). In contrast, shotgun protein sequencing techniques enable quantification of protein abundance and subsequent functional annotation (44, 45). The inverse of functional diversity is functional redundancy, which describes that organisms share overlapping ecological functions. Recent studies have started to explore the redundancy of the functional capacity in the gut microbiome (46). However, the extent of redundancy of expressed functions, and how such redundancy contributes to functional compensation between species remain unexplored. Metaproteomics can add a helpful dimension to such studies to reveal the relationship between the redundancy of the functional capacity, as well as the actual functional compensation that happens under different conditions.

##### Matter and Energy Are Basis for Ecosystem Models

Matter and/or energy are often the basis for community and ecosystem models (26, 47), from population dynamics to mass and energy budgets models of a microbial community. For example, a simple model that describes the mucosal and luminal microbial communities at the MLI can be established using a finite element method based on biomass and nutrient flow in/out the finite element and between the mucosal and luminal communities (Fig. 1*D* and supplemental Information). The model consists of a luminal microbial community (size: *C_2_*), a mucosal microbial community (size: *C_1_*), a nutrient pool of the gut lumen (size: *N*), and a nutrient pool of the mucus (size: *M_u_*). The biomass dynamics in microbial communities C_1_ and C_2_ could then be presented as a function of biomass increase (as functions of nutrient sizes), biomass exchange between *C_1_* and *C_2_* (syntrophic interactions and bacterial dispersals), metabolism and mortality, and biomass output. Experimental data are required to establish microbial growth models of the microbial communities and sizes of nutrient pools. Ecological properties of the microbial communities (*e.g.* carrying capacity, intrinsic rate of growth, etc.) could then be estimated by the models to understand the microbiome dynamics (supplemental Information). A few studies have been performed to describe the ecological dynamics of our gut microbiota. Stein *et al.* have described microbiota dynamics using generalized Lotka–Volterra model with the addition of external perturbations (48). Subsequently, the Microbial Dynamical Systems INference Engine (MDSINE), an open source software package has been developed to facilitate its application (49). However, because of the limitation of relative abundance measurements (for example, 16S rRNA or metagenomics sequencing), a measurement for overall microbial biomass was needed in addition to relative abundances (50). For example, in the reported study, universal 16S rRNA quantitative PCR (qPCR) was used to measure the microbiome biomass (49). Taxonomic composition estimated by metagenomics and metaproteomics are considered generally comparable (42, 51). However, because different microbial members can differ by several orders of magnitude in biomass (52), other studies have shown that metaproteomics is more accurate to assess biomass contributions of organisms in microbial communities (53, 54). Metaproteomics have been used to build growth functions of an *in vitro* gut microbiome (55). In terms of matter and energy flow, metaproteomics-based technologies such as protein-SIF are developed to determine carbon and energy sources and metabolic pathways of individual species within a microbial community (56).

##### Site-Specific Insight is Required for Host-Microbiome Interaction

Moreover, comprehensive use of proteomics and metaproteomics can provide site-specific functional insight into host-microbiome interaction. Many studies on the gut microbiome are based on fecal samples, which are not representative of the microbiota at different intestinal regions. Studies have shown that combining site-specific sampling with proteomics and metaproteomics enables more comprehensive understanding of the MLI. Li *et al.* performed microgeographic studies on the mucosal-luminal interface in IBD and nonIBD subjects through collecting mucosal lavage samples on 1 cm diameter sites from different intestinal regions, and separately analyzed the bacterial pellets and soluble components in each sample using metaproteomics (37, 38). The studies identified proteins and functional protein networks that were biogeographically associated to different colon regions (32), and spatially-associated protein expressions related to IBD. Similarly, Presley *et al.* used endoscopic saline-lavage sampling to collect MLI samples from various regions of the intestine and examined the bacterial rRNA gene and metaproteomics composition in ulcerative colitis (UC), Crohn's disease (CD) and nonIBD individuals. Results showed greater difference of phylotypes and protein expressions between disease types in the sigmoid colon than in the cecum (40). In terms of host proteins, Deeke *et al.* analyzed the host proteome of MLI aspirates from the ascending colon (AC) and descending colon (DC) of nonIBD and IBD children for the discovery of biomarkers. Multivariate analysis between IBD and nonIBD samples discovered higher number of differential proteins in the DC than in the AC (57). The above studies achieved site-specific proteomics or metaproteomics analysis, and a most recent approach comprehensively analyzed human-derived proteins, metaproteome of bacteria, fungi, archaea and viruses, as well as extracellular vesicles (EVs) from the AC, DC, or terminal ileum (TI) of IBD patients, and revealed the role of EVs in host-microbiome interactions in IBD (39).

Proteomics and metaproteomics bring added dimensions, such as matter, energy, and functional dimensions, to studying the MLI ecology. Moreover, they provide site-specific insights on host, microbes, viruses, and extracellular proteins involved in host-microbiome interactions.

## PROTEOMICS AND METAPROTEOMICS APPROACHES TO DISSECT THE MLI ECOLOGY

##### Isolating Different MLI Compartments

There are various sources and types of samples that are used to study the MLI, *e.g.* patients that are undergoing colonoscopic diagnosis, animal models, and *in vitro* systems. The host proteome portion can be obtained through human colon biopsy, animal colonic segments, or by collecting the host cell portions from an *in vitro* host-microbiome model. For the metaproteome portions, samples need to be properly processed to isolate the mucosal and luminal components. In studies on human subjects, stool/luminal aspirate and mucosal biopsy are used to separate the mucosal and luminal portions (34, 35, 39, 58, 59). Mucosal and luminal content of animal colon are often separated by washing off the luminal content and then scraping off the mucosal content (36, 60, 61). Besides *in vivo* studies, there are also *in vitro* systems that enable studying different components of the MLI. A mucosal-simulator of human intestinal microbial ecosystem (M-SHIME) has been developed to simulate the mucosal gut microbiota by creating a niche using microcosms submerged in mucin agar and combined in a polyethylene netting (60, 62). Furthermore, researchers have been developing *in vitro* systems to study host-microbiome interactions at the MLI. A microfluidics-based model for studying human–microbial cross-talk (HuMiX) has been designed for representative co-culture of human epithelial cells with gastrointestinal microbiota (63, 64). This model involves a mucin-coated nanoporous membrane to provide a surface niche for the mucosal community. Recent development of the gut-on-a-chip models has achieved epithelial villus growth and lineage-dependent cyto-differentiation. The cultured intestinal epithelium can secrete mucus and thus provides a barrier function (33, 65, 66). Latest study showed that this model is also able to sustain a complex human intestinal microbiome *in vitro* (67). These models provide reproducible systems of host-microbiome interface in which host and mucosal, luminal compartments are isolatable for downstream analysis.

##### Capturing Desired Arrays of Proteins

Following sample acquisition, selection of proteomic sample preparation method is important for capturing the desired arrays of proteins (Fig. 1*E*). Microbial community sample can be highly complex because of the existence of virome and exoproteins (microbial & host extracellular proteins) in addition to the presence of diverse bacteria and fungi with differing types of envelopes. Most widely adopted protein extraction procedure involves a microbial cell washing step, followed by microbial cell lysis and extraction of total proteins. However, conventional protein extraction methods can eliminate important components of host-microbiome interaction, such as the virome and exosome. Exosome proteins can be extracted by ultracentrifugation of the filtrate of debris- and bacteria-depleted supernatant (39). Virome proteins can be isolated by enrichment of viral-like particles where different type of enriching techniques may apply (68). In addition, conventional protein extraction from microbial cell pellets can miss the measurement of low-abundance species. Differential cell lysis approaches can be applied when necessary to selectively enrich Gram-negative bacteria and Gram-positive bacteria in a sample (69). Furthermore, deep metaproteomics can be achieved by sample fractionation before MS analysis. Through a comprehensive and deep proteomics-metaproteomics approach, Zhang *et al.* have realized quantification of microbial metaproteome, human proteome, and extracellular vesicles in individual MLI aspirate samples (39).

##### Targeting Specific Pathways

In addition to isolation and enrichment of different compartments at the MLI, labeling of targeted proteins can enable enrichment and quantification of diverse functional mechanisms in combination with proteomics and metaproteomics. In contrast to unbiased ‘omics‘-based approaches, activity-based probes (ABPs) can be designed based on the substrates of interest (70), and ABP-labeled proteomes can be enriched based on fluorophore tag and gel electrophoresis, or by affinity purification using an affinity tag. The approach facilitates quantification of functionally active proteoforms of interested enzymes, which could have low abundances in a complicated protein mixture. Studies have used ABPs to target a wide range of enzymes such as hydrolases, proteases, kinases, phosphatases, and glycosidases. Recently, researchers started to apply ABP-based metaproteomics in gut microbiome studies. Mayers *et al.* have used a BioGlyCMK probe to target the subset of cysteine-based proteases in murine gut metaproteome and found that several proteases and hydrolases overrepresented in the IBD mice compared with the control (71). Using a Ch-AOMK probe targeting bile salt hydrolases (BSH), Parasar *et al.* have observed changes in gut microbiome-associated BSH activity in IBD mice, whereas these changes do not correlate with changes in gene abundance (72). Jariwala *et al.* have applied cyclophellitol-based probes to identify β-glucuronidases that are related to promotion of drug toxicity in human fecal samples (73). β-glucuronidases belong to glycoside hydrolases (GHs), the biggest class in Carbohydrate Active enZymes (CAZymes) (74). CAZymes are widely expressed by gut microbes to breakdown carbohydrates derived from both diet and the host, such as host-derived mucins (glycoproteins), oligosaccharides, and dietary fibers, etc. CAZymes in the gut are highly diverse because of substrate specificity, the application of a variety of CAZyme-ABPs is promising in determining the strategies of carbohydrate-degradation in the gut microbiome.

##### Tracking Nutrient Flows

Metabolic labeling of active microbial species can be achieved by protein-based stable isotope probing (SIP) techniques, which starts with supplying growth substrates labeled with heavy isotopes such as ^13^C, ^15^N, ^18^O, ^2^D, and ^33/34/36^S that can constitute the protein molecules in a live community (56, 75, 76, 77, 78, 79). Owing to the sensitivity of LC–MS/MS techniques in detecting heavy isotopes, degree of incorporation of these heavy isotopes into proteins can be determined accurately on the peptide level. Therefore, SIP in combination with metaproteomics provides in-depth characterization of key players in the microbiome by tracking down the uptake, degradation, cross-feeding and conversion of a labeled substrate (Fig. 1*F*). For example, in an environmental microbiome study, protein-SIP has been used to observe carbon flow and functional interactions within the benzene-degrading, sulfate-reducing community (78). Kleiner *et al.* have developed a direct protein stable isotope fingerprint (SIF) technique and software packages to track the consumption of environmental carbon sources by microbial species in communities through determining their stable carbon isotope ratios (δ^13^C) (56). This approach can determine the nutrient flow of specific carbon source in individual species and subsequent pathways to assimilate the carbon source. In addition to carbon, nitrogen has been used to generate labeled gut microbial community both through *in vitro* culturing and through feeding animals using ^15^N-based substrates (71, 79). By feeding mice using ^15^N spirulina diet, Mayers *et al.* showed that 95% ^15^N incorporation in peptides was attained within 4 weeks of feeding. *In vitro* culturing can achieve more rapid and efficient ^15^N incorporation. Zhang *et al.* have found that following a SILAMi approach, >95% ^15^N enrichment in an *in vitro* gut microbiome can be achieved within 3 days of culture (79). This metabolically stable isotopic labeling of microbiota was aimed as an internal standard for quantitative metaproteomics, yet it may be extendable to trace the nutrient flow of nitrogen sources in the environment between microbial species when observed through the time course.

## PERSPECTIVES

**Fig. 2:**
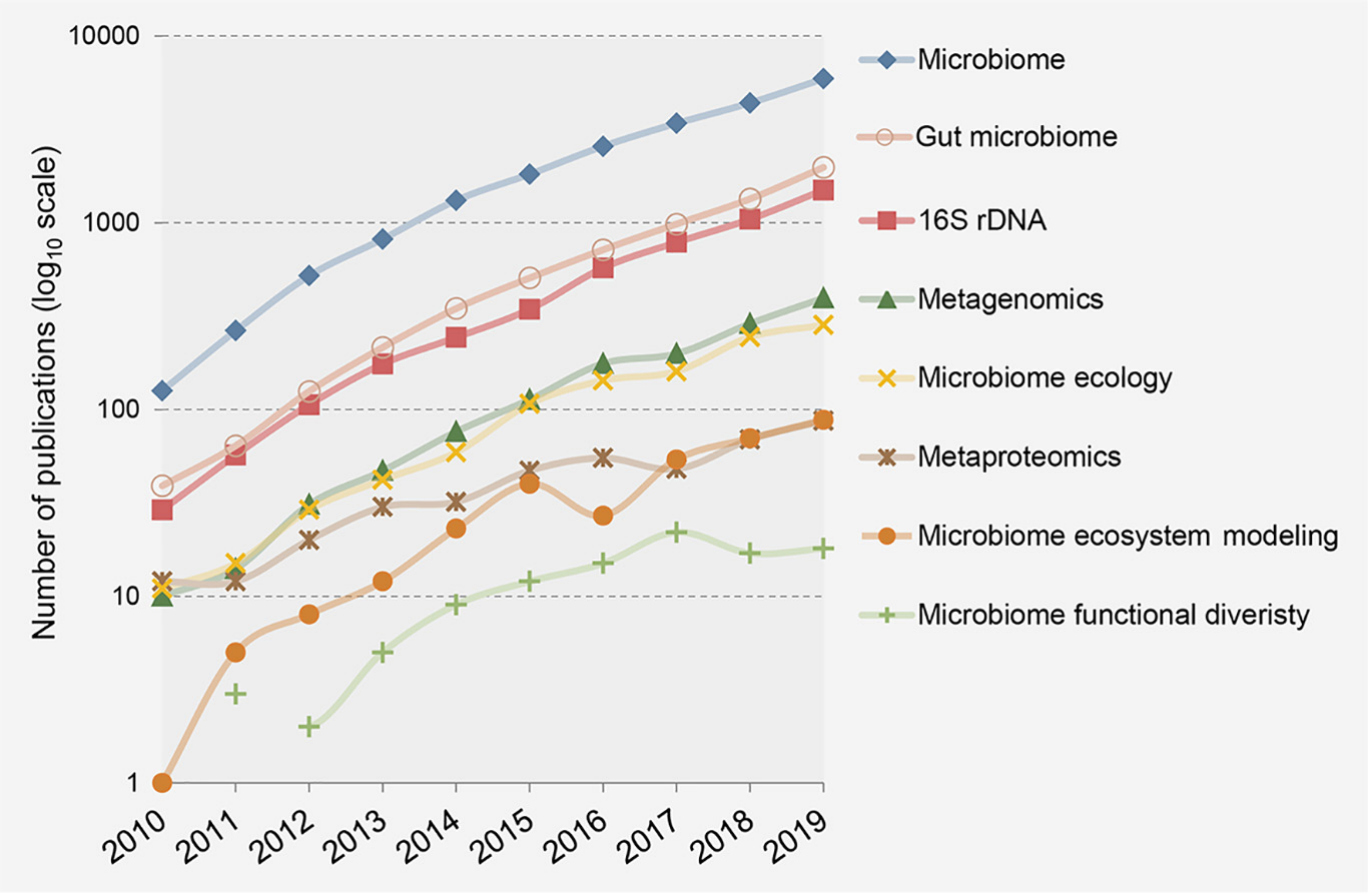
Number of publications in the recent decade corresponding to different keywords (PubMed).

Although the number of gut microbiome study grew exponentially over the past decade (Fig. 2), most studies are still focused on taxonomic composition and functional capacities based on 16S and metagenomics. Metaproteomics is emerging with an average increase of 30% more publications per year, helping to expand our understanding of the microbiome functional ecology. Interestingly, the number of microbiome publications that involves a discussion on “ecology” has been increasing with comparable number to metagenomics. However, in comparison, significantly less effort has been directed to practical ecosystem modeling, which mathematically describes the organization mechanism of our complex gut ecosystem. Furthermore, more attention needs to be paid to the role of functional diversity in our gut ecosystem.

Mathematically describing the dynamics of microbial communities have long been performed in other disciplines such as environmental and agricultural microbiomes. Similarly, it is important to explicitly describe the interaction between microbial species as well as between host and gut microbiota, so as to better understand our health and disease. Scientists engaged in modeling the gut microbiota found that it is important to build models based on function- and biomass- experimental data in addition to genomic sequencing (49). It has been suggested that proteomics and metaproteomics are important approaches to generate mass and functional arrays based on proteins. In addition, to describe an ecosystem, we need to consider the spatiotemporal property of our host-microbiome ecosystem. Microgeographic differentiation and identification of proteins from different biological kingdom can be readily achieved by proteomics and metaproteomics sampling and enrichment techniques, and thus facilitates comprehensive data representation of the MLI. Finally, proteomics and metaproteomics in combination with *in vitro* models will provide efficient, reproducible and objective solution to future MLI ecology studies.

It is notable that the technologies still face several challenges. Sample preparation remains complicated and usually many days are needed to perform protein extraction, digestion to desalting. Although rapid high-throughput proteomics and metaproteomics techniques have been developed, experiments are expensive and extensive experimental and bioinformatics expertise are still needed. In addition, current metaproteomics has a limited sequencing depth because of the high complexity of the gut microbiome. Nevertheless, experimental, instrumental and bioinformatic techniques for metaproteomics are evolving rapidly, and we expect more in-depth characterization of the gut metaproteome and broader applications.

## CONCLUSION

We are still at the early stage of exploring the ecosystem principles that maintain the homeostasis of our gut microbial community and host-microbiome relationship. Recent development of proteomics and metaproteomics technologies can provide promising contribution to studying spatiotemporal host-microbiome interaction at the MLI. Important approaches facilitating such studies include isolation strategies for different MLI components, enrichment methods to obtain designated array of proteins, probing for specific pathways, isotopic labeling for tracking nutrient flow, and the use of *in vitro* MLI models. Therefore proteomics and metaproteomics, based on properly selected protocols, can provide information on functional diversity, matter and energy flow, and site-specific insights that are suitable for mathematical modeling of the MLI ecosystem.
