# Supplementary material for: Proteomics and Metaproteomics Add Functional, Taxonomic and Biomass Dimensions to Modeling the Ecosystem at the Mucosal-luminal Interface
Source: Mol Cell Proteomics. 2020 Nov 25;19(9):1409–17. doi: 10.1074/mcp.R120.002051 (PMC8143649; doi:10.1074/mcp.R120.002051)
Supplement: Supplementary file 1 [file mmc1.pdf]

Supplementary Information

A mathematical model describing the MLI ecosystem based on mass-flow:

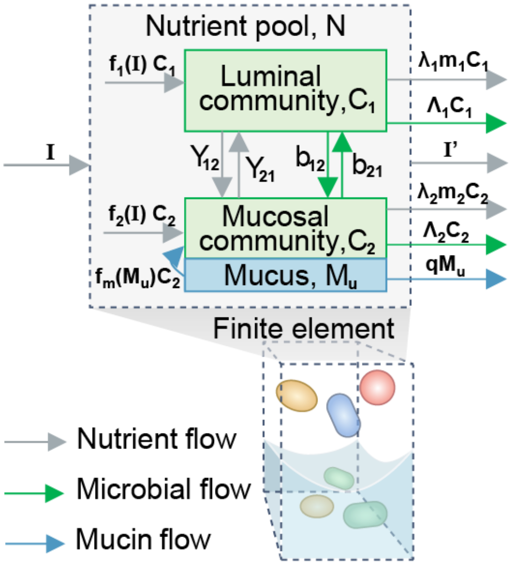

In order to describe the gut ecosystem, a finite element is taken from the MLI. This finite element includes a luminal microbial community (size: C_2), a mucosal microbial community (size: C_1), a nutrient pool of the gut lumen (size: N), and a nutrient pool of the mucus (size: M_u).

Other parameters shown in the figure are:

- I, constant input of nutrient per unit time
- I’, un-used nutrient per unit time (parts that are not involved in metabolism)
- $f_i(I)$, functional response of community i to nutrient availability
- λ_i , fraction of nutrient lost from community i
- m_i , release of nutrient from community i due to metabolism & mortality

- Y_{ij} syntrophic interactions in which C_i provides nutrients to C_j
- Λ_i, fraction of bacterial loss due to continuous flow in community i
- Λ, total bacterial loss due to continuous flow
- b_{ij}, bacterial dispersal from C_i to C_j

where, i, j ∈ [1, 2], i≠j

System dynamics can be described using the following equation:

i. Bacterial communities: biomass dynamics

$$\frac{dC_1}{dt} = f_1(I)C_1 + (Y_{21} - Y_{12}) + (b_{21} - b_{12}) - \lambda_1 m_1 C_1 - \Lambda_1 C_1 \quad (1)$$

$$\frac{dC_2}{dt} = f_2(I)C_2 + f_m(M_u)C_2 + (Y_{12} - Y_{21}) + (b_{12} - b_{21}) - \lambda_2 m_2 C_2 - \Lambda_2 C_2 \quad (2)$$

Increase of biomass	Exchange of biomass	Metabolism & mortality	Biomass output

ii. Nutrient pool dynamics

$$\frac{dN}{dt} = I - I' - \lambda_1 m_1 C_1 - \Lambda_1 C_1 - \lambda_2 m_2 C_2 - \Lambda_2 C_2 \quad (3)$$

iii. Growth of microbial communities

Assume logistic growth of both communities

Increase of biomass – metabolism & mortality:

$$f_1(I)C_1 - \lambda_1 m_1 C_1 = r_1 C_1 (1 - \frac{C_1}{K_1}) \quad (4)$$

$$f_2(I)C_2 + f_m(M_u)C_2 - \lambda_2 m_2 C_2 = r_2 C_2 (1 - \frac{C_2}{K_2}) \quad (5)$$

Where, r_i intrinstic rate of natural growth; K_i carrying capacity of the system

At equilibrium:

$$\frac{dC_i}{dt} = 0 \quad (6)$$

hence

$$C_i^* = K_i \frac{r_i - \Lambda_i \pm \sqrt{(r_i - \Lambda_i)^2 + 4\frac{r_i}{K_i}(\Delta Y_{ij} + \Delta b_{ij})}}{2r_i} \quad (7)$$

Thus, if assume logistic growth of both mucosal and luminal communities, an equilibrium may be achieved in relation to ecosystem carrying capacity (nutrients from food and mucus), intrinsic rate of growth (microbial community activities), bacterial loss ratio (bowel movement), as well as syntrophic interactions and bacterial dispersal between the two communities. Assumptions/parameters need to be improved/determined by experiments. Metaproteomics will be an adequate tool for obtaining the data.
